# Long-term results and quality of life after stapled hemorrhoidopexy vs Doppler-guided HAL-RAR: a propensity score matching analysis

**DOI:** 10.1007/s00384-024-04603-0

**Published:** 2024-02-22

**Authors:** Sara Lauricella, Dario Palmisano, Francesco Brucchi, Domenico Agoglitta, Massimiliano Fiume, Luca Bottero, Giuseppe Faillace

**Affiliations:** 1 Department of General Surgery, ASST-Nord Milano, Sesto San Giovanni City Hospital, Sesto San Giovanni, Lombardia, Italy; 2 General Surgery Residency Program, University of Milan-Bicocca, Milan, MI Italy; 3 Department of General Surgery, Bassini Hospital, Via Massimo Gorki 50, 20092 Lombardia, Cinisello BalsamoMI Italy; 4 General and Laparoscopic Surgery Residency Program, University of Milan, Milan, MI Italy

**Keywords:** Hemorrhoidopexy, Doppler-guided HAL-RAR, Quality of life

## Abstract

**Aim:**

The study aimed to evaluate and compare the short and long-term outcomes of doppler-guided (DG) hemorrhoidal artery ligation and rectoanal repair (HAL-RAR) using a wireless-doppler-guided probe and stapled hemorrhoidopexy (SH) for treatment of II–III hemorrhoids.

**Methods:**

This cohort study included patients who underwent HAL-RAR (*n* = 89) or SH (*n* = 174) for grade II-III hemorrhoids between January 2020 and December 2021. After propensity score matching at a 1:1 ratio, 76 patients for each group were analyzed. Short and long-term outcomes were collected. Pain was measured using a Visual Analogue Scale (VAS) at POD1, POD 10, 1 month, and 6 months after surgery. The enrolled patients completed the Hemorrhoidal Disease Symptom Score and Short Health ScaleHD quality of life (HDSS/SHS QoL) questionnaire preoperatively and during a regular follow-up visit at 24 months after surgery.

**Results:**

Groups exhibited comparable overall postoperative complication rates (23% HAL-RAR/ 21% SH; *p* = 0.295). Postoperative pain via VAS showed median scores of 4, 3, 1, 1 for HAL-RAR and 6, 4, 2, 1 for SH at POD1, POD10, 1 month, and 6 months, respectively (*p* =  < 0.001, 0.004, 0.025, 0.019). At a median follow-up of 12 months, the recurrence rate was 10.5% in the HAL-RAR group and 9.2% in the SH group (*p* = 0.785), respectively. At 24 months, 15.7% of HAL-RAR patients and 19.7% of SH patients remained symptomatic (*p* = 0.223). Median post-op QoL index was 1 (HAL-RAR) and 0.92 (SH), *p* = 0.036.

**Conclusions:**

HAL-RAR is a safe and feasible technique in treating grade II-III hemorrhoids showing better outcomes in terms of postoperative pain and QoL.

**Significance:**

This paper adds a new perspective in comparing the HAL-RAR and SH, focusing the attention on the patients and not surgical techniques. A long and difficult follow-up was completed to fully understand the long-term results and the impact on the QoL of the patients who underwent these procedures.

## Introduction

Hemorrhoidal disease (HD) is the most common proctological disorder with an estimated prevalence of 4.4% and one of the most frequent causes of severe acute lower gastrointestinal bleeding [1, 2].

Several therapeutic options have been described for the treatment of hemorrhoids ranging from conservative treatments (changes in eating habits, phlebotonics, topical treatment) and outpatient treatments (rubber band ligation, injection sclerotherapy, infrared coagulation) to non-excisional surgical procedures (stapled hemorrhoidopexy, transanal hemorrhoidal dearterialization) and excisional procedures (open or closed hemorrhoidectomy) [1].

Post-surgical pain is one of the most common and disabling complications after surgery with an important negative impact on a patient’s quality of life [3]. Thus, the concept of treatment has evolved with less invasive techniques that aim to reduce postoperative pain and discomfort.

Doppler-guided (DG) transanal hemorrhoidal artery ligation (HAL) was initially described by Moringa on 116 consecutive patients with internal hemorrhoids, and the results improved when combined with mucopexy of the prolapsed hemorrhoidal tissue (RAR = Recto Anal Repair). HAL-RAR using specifically designed equipment provides an excellent visualization of the different hemorrhoidal pedicles and the lower rectum. This is of paramount importance for the correct ultrasound-guided placement of arterial ligatures which reduces the arterial hyperflow in the anal vascular cushions and the mucopexy.

The new technique has proved to be feasible and effective, showing encouraging results in the control of symptoms even for the treatment of advanced hemorrhoidal disease [2]. Two studies reported a significantly low incidence of postoperative pain and good control of hemorrhoidal symptoms in more than 85% of cases after a mean follow-up of 34 and 12 months, respectively. The overall postoperative complication rate was 9%. No major complications were encountered, and no patient required revision surgery [4].

Additionally, results from the LigaLongo Trial comparing HAL-RAR (*n* = 193) and circular stapled hemorrhoidopexy (*n* = 184) showed similar postoperative morbidity and surgery outcome at 1 year regardless of the type of the device used for grade II-III hemorrhoids. These findings suggest that the device type has little impact on the results when used by well-trained colorectal surgeons [5].

The purpose of the present study is to evaluate and compare the short and long-term outcomes in terms of adverse events, postoperative pain, recurrence rate, and quality of life (QoL) of patients with grade II-III hemorrhoids according to the type of device used.

## Method

The cohort study included patients with grade II-III hemorrhoids undergoing surgery at the Department of General Surgery, ASST-Nord Milano, between January 2020 (since the introduction of the new device) and December 2021. The inclusion criteria were as follows: age ≥ 18 years, patients with a diagnosis of grade II-III hemorrhoids based on Goligher’s classification [6], and failure to conservative treatments. Exclusion criteria were as follows: degree IV hemorrhoids, patients with previous rectal radiation, inflammatory bowel disease, immunocompromised or with co-existent anal pathologies such as anal fistula, perianal abscess, anal cancer, concomitant rectocele, and if follow-up information was incomplete or missing. Patients who underwent previous hemorrhoid-related surgery were also excluded.

After obtaining institutional ethics review board approval, all epidemiological data were prospectively collected by the hospital information system, and a large database was set up.

Data included patient characteristics (age, gender, BMI, associated comorbidities, ASA score), disease characteristics (grade hemorrhoidal disease), surgical technique (SH vs HAL-RAR, operating time, intraoperative device dysfunction), length of hospital stay (LOS), “early” (< 30 days) postoperative complications (pain, bleeding, emergency reoperation, early fecal urgency, tenesmus, thrombosis, fissure, urinary retention), and long-term complications (anal stenosis, soiling, dyschezia, urgency, fecal incontinence, pruritus, skin tags, recurrence, readmissions, redo surgery). The choice of surgical technique focused on a broader shared-making process. Specifically, the decision on the type of surgery depended on many factors including patients’ personal preferences, surgical expertise, and the presence of a circumferential or asymmetric muco-hemorrhoidal prolapse. The patients were fully informed about the pros and cons of the available surgical options. Complications were classified using the Clavien Dindo classification [7]. A visual analogue scale (VAS) was used to measure pain, ranging from 0 (no pain) to 10 (worst pain imaginable).

Patients who underwent HAL-RAR were recruited to the HAL-RAR group, and patients who underwent SH were recruited to the SH group and selected by 1:1 propensity score matching (PSM), matched using age, ASA score, BMI, Hemorrhoidal Disease (HD) grade, and preoperative symptoms as covariates in multivariate analysis; logistic regression was used to calculate propensity scores, and the treatment strategy was the dependent variable. In the HAL-RAR group, we used a wireless-Doppler guided probe (HALO™ Trilogy-A.M.I. Ltd, Feldkirch, Austria) which combines hemorrhoidal artery ligation and mucopexy of the prolapsing mucosa. HAL-RAR with the flexibility of wireless technology is easily manageable by surgeons and offers an absolute freedom to maneuver the unit and the attached probe. In the SH group, we used a new high-volume stapling device Chex ™ CPH34HV (Frankenman International Ltd. Sheung Wan, Hong Kong).

Preoperative, intraoperative, and postoperative data had been prospectively collected.

All patients were followed up for at least 24 months. Outpatient follow-up was carried out at 7 days, 1 month, 6 months, 12 months, and 24 months after discharge.

The enrolled patients completed the HDSS (Hemorrhoidal Disease Symptom Score) based on the 5 primary symptoms (pain, itching, bleeding, soiling, and prolapse) and the SHS (Short Health Scale) questionnaire, a subjective health assessment instrument [8–10] preoperatively and during a regular follow-up visit at 24 months after surgery.

All data analyses were performed using Stata MP/15.1 (StataCorp, Lakeway Dr, USA).

Quantitative data were summarized using mean, standard deviation, median, and range. Qualitative data were summarized using frequencies and percentages. Numerical variables regarding preoperative and 24-month QoL were compared using univariate analysis (the Wilcoxon signed-rank test) when appropriate.

## Results

The cohort study included 263 patients of which 174 (66.16%) underwent high-volume SH and 89 (33.84%) underwent HAL-RAR using a wireless-Doppler guided probe. Multivariate analysis, when comparing SH vs HAL-RAR, identified significant imbalances in age (*p* = 0.061), HD grade (*p* = 0.043), ASA score (*p* = 0.006), BMI (*p* = 0.007), and preoperative symptoms such as bleeding and anal pain (*p* = 0.001). After propensity score matching (PSM), 76 matched pairs of patients were created. All covariate imbalances were alleviated. Patients’ baseline characteristics before and after matching are summarized in Table 1.

**Table 1:** Baseline characteristics of the two groups before and after PSM

	**Unadjusted**			**Propensity analysis**		
	**HAL-RAR (*n* = 89)**	**SH (*n* = 174)**	***p* value**	**HAL-RAR (*n* = 76)**	**SH (*n* = 76)**	***p* value**
**Male/female (*n*)**	52/37	96/78	0.614	42/34	47/29	0.410
**Age**	46 (38–52)	57 (32–71)	0.020	52 (46–61)	53 (51–61)	0.076
**ASA score I/II/III (*n*)**	32/43/14	51/56/67	0.006	17/32/27	13/41/22	0.340
**BMI**	24.6 (22.55–27.65)	27.7 (26.1–29.9)	0.007	25.8 (23.6–26.4)	26.5 (25.4–28.2)	0.123
Preoperative symptoms (B/P/Bo/N)*	17/28/13/31	36/22/21/95	0.001	16/23/9/28	18/21/7/30	0.912
**Preoperative VAS**	2 (1–3)	3 (2–4)	0.230	3 (2–3)	3 (2–3)	0.170
**HD grade II/III (*n*)**	76/13	83/91	0.043	63/13	61/15	0.675

Legend 1: B* bleeding, P* pain, Bo* both symptoms, N* no symptoms

There was no significant difference in the overall complication rate between the SH and HAL-RAR groups after PSM (SH 35% vs HAL-RAR 35%, *p* = 0.258). Early (< 30 days) complication rate was 23% in the SH group and 21% in the HAL-RAR group (*p* = 0.295). Specifically, when SH was compared to the HAL-RAR group, dyschezia occurred in 6.5% vs 4% (*p* = 0.953) of cases, soling in 2.5% vs 4% (*p* = 0.886), itching in 4% vs 1.5% (*p* = 0.694), and anal pain in 1.5% vs 2.5% (*p* = 0.031). No cases of early fecal urgency, thrombosis, fissure, or urinary retention have been observed.

The median operating time was 25 min (IQR = 22–29) for HAL-RAR and 27 min (IQR = 25–31) for the SH group. After PSM, the median LOS was comparable for the two groups. No patients required hospitalization. All patients were discharged the day after the procedure.

Pain was prospectively measured with a VAS scale at 1, 7 days, 1 month, and 6 months after surgery. The median VAS values at the time mentioned above points were 4/3/1/1 and 6/4/2/1 for the HAL-RAR and SH group, respectively. (*p* =  > 0.001, 0.004, 0.025, 0.019).

After PSM, the overall recurrence rate was 10.5% and 9.2% in the HAL-RAR vs SH group (*p* = 0.785), respectively. The mean time to recurrence was 12 months for both groups. No patient required blood transfusions. In the HAL-RAR group, 3 patients required excisional hemorrhoidectomy, and one patient had a redo HAL-RAR. One patient had mild to moderate postoperative anal pain which was treated with oral paracetamol and ketorolac tromethamine. Three cases of recurrence hemorrhoid after stapled hemorrhoidopexy were managed by redo hemorrhoidopexy. One patient complained of mild tenesmus, but he well-tolerated the therapy with good results; tenesmus disappeared in a few weeks, and the patients did not require hospitalization. No recurrence was reported. All patients who underwent redo surgery were satisfied with the results achieved. In our series, no cases of anal stenosis, soiling, dyschezia, urgency, fecal incontinence, pruritus, or skin tags have been observed (Table 2).

**Table 2:** Intervention, complications, and recurrence before and after PSM

	**Unadjusted**			**Propensity analysis**		
	**HAL-RAR (*n* = 89)**	**SH (*n* = 174)**	***p* value**	**HAL-RAR (*n* = 76)**	**SH (*n* = 76)**	***p* value**
**Overall complications at 30 days**	25%(*n* = 22)	22% (*n* = 38)	< 0.001	23% (*n* = 17)	21% (*n* = 16)	0.295
**Dyschezia**	9.5% (*n* = 8)	5% (*n* = 9)	0.967	6.5% (*n* = 5)	4% (*n* = 3)	0.953
**Soiling**	5.5% (*n* = 5)	4% (*n* = 7)	0.853	2.5% (n = 2)	4% (*n* = 3)	0.886
**Itching**	6% (*n* = 5)	7% (*n* = 15)	0.628	4% (*n* = 3)	1.5% (*n* = 1)	0.694
**Anal pain**	4% (*n* = 3)	6% (*n* = 10)	0.912	1.5% (*n* = 1)	2.5% (*n* = 2)	0.031
**Operating time (median/IQR)**	27/21–33	33/24–37	0.878	25/22–29	27/25–31	0.342
**LOS (in days)**	1	2	\	2	2	\
**Recurrence (Y/N)**	9/80	11/163	0.272	8/68	7/69	0.785

Legend 2: *D *dyschezia, *S *soiling, *I *itching, *A *anal pain, *N *none

After 6 months of follow-up, 6 dearterialization patients and 11 hemorrhoidopexy patients experienced recurrent symptoms, mainly anal pain (*p* = 0.854). Additionally, at 6 months after surgery, 9 patients experienced bleeding in the HAL-RAR group and 11 in the SH group. No patient required hospitalization or blood transfusion.

After PSM one-hundred and twenty-six patients (*n* = 126) attended the 2-year follow-up.

The median preoperative QoL was 0.81 for the HAL-RAR group and 0.80 for the SH group (*p* = 0.699). At 24 months, 81/89 HAL-RAR and 112/174 SH patients completed the follow-up. The QOL indexes increased to 1 for the first group and 0.92 for the second one (*p* = 0.472).

Table 3 summarizes the surgical outcomes.

**Table 3:** Evolution of quality of life before and after PSM

	**Unadjusted**			**Propensity analysis**		
	**HAL-RAR (*n* = 89)**	**SH (*n* = 174)**	***p* value**	**HAL-RAR (*n* = 76)**	**SH (*n* = 76)**	***p* value**
**Postop. pain at 1 day**	5 (4–6)	7 (4–8)	< 0.001	4 (4–6)	6 (5–7)	< 0.001
**Postop. pain at 7 days**	4 (3–5)	5 (3–7)	0.007	3 (3–4)	4 (3–4)	0.004
**Postop. pain at 1 month**	1 (1–2)	2 (1–2)	0.075	1 (1–1)	2 (1–1)	0.025
**Postop. pain at 6 months**	1 (1–1)	1 (1–1)	0.048	1 (1–1)	1 (1–1)	0.019
**Bleeding at 6 months Y/N**	13/76	24/150	0.857	9/67	11/66	0.854
**2 years follow-up**	81	112	/	65	61	**/**
**Preop. QoL Index**	0.79 (0.72–0.89)	0.77 (0.70–0.83)	0.673	0.81 (0.76–0.86)	0.80 (0.75–0.84)	0.699
**24 months QoL Index**	1 (0.82–1)	0.88 (0.77–0.94)	0.842	1 (0.85–1)	0.92 (0.83–0.95)	0.036

Legend 3: *Y *yes; *N *no

## Discussion

HD is the most frequent proctological disorder that negatively impacts patients’ quality of life. As postoperative pain is the major concern for patients undergoing proctological procedures, surgery has evolved over the last decade with less invasive, continence-preserving techniques while ensuring limited rates of complications and recurrence.

Although the classic excisional techniques are associated with low recurrence rates compared to non-excisional procedures, they seem more painful [1]. The pain may be due to excessive tissue resection and aggressive mucosal management which can irritate the perianal nerves [11].

Conversely, the non-excisional surgical procedures have the advantage over excisional techniques of less associated local trauma, rapid recovery, and less intense postoperative pain but seem to be associated with higher recurrence rates [1, 12].

In our series, a significant decrease in pain was observed in patients undergoing HAL-RAR using a wireless-Doppler guided probe during the first 30 days after surgery. HAL-RAR is a minimally invasive technique that is conceptually painless for patients since all maneuvers and sutures are above the dentate line. The interruption of the blood supply to the hemorrhoidal venous cushions without interfering with anal anatomy results in faster wound healing and patient recovery [13]. Of note, in our experience from day 30, the pain in patients who underwent high-volume SH gradually decreased to equalize both techniques by 6 months (1 vs.1; *p* = 0.019).

Studies examining both procedures [11, 14] underline the connection between preoperative and postoperative pain: the lower the preoperative pain, the lower the postoperative one would be. To equate both groups of patients, we decided to stratify them with a propensity score matching analysis. The use of PSM has been acknowledged as a reliable tool for mitigating selection bias in non-randomized studies and minimizing heterogeneity within study groups when comparing outcomes of interest [15].

Postoperative bleeding represents one of the most disabling symptoms especially when persisting after surgery. In our experience, 10.5% of cases in the HAL-RAR and 9.2% in the SH group experienced postoperative bleeding during the first 30 days. Results from Karkalemis et al. revealed that [13] one of the key advantages of the HAL-RAR procedure is the almost total absence of blood loss during and after surgery due to the accurate artery ligation provided by the intraoperative Doppler. The higher early postoperative bleeding rate of the SH may be due to the stapled resection. Limiting the procedure to the mucosal tissue is not always achievable. If the stapling involves the submucosal/muscular layers, which have more vessels than the superficial layer, there is an increased chance of post-procedure bleeding from resected vessels.

At 6 months follow-up bleeding recurred in 9 (11.8%) and 11 patients (14.4%), respectively (HAL-RAR vs SH group, *p* = 0.854). However, 1 year after surgery, bleeding rates were comparable between the two groups of patients. Symeonidis et al. described similar findings in terms of bleeding [16] in their retrospective study on 64 patients who underwent HAL-RAR with mucopexy.

Recurrence is one of the most important outcomes in hemorrhoidal management, and the most examined item when a new gold standard treatment is analyzed [11]. Recurrence occurred in 8 (10.5%) patients in the HAL-RAR and 7 (9.2%) in the SH group. Before PSM, only 6.3% of SH patients had recurrence (vs 10.1% in the HAL-RAR group). However, neither before nor after PSM do these results attain statistical significance. Many studies corroborate these findings, in particular the one from Xu et al. [17]. In the latter, it is highlighted that SH has a lower recurrence rate due to a more radical mucosal approach, especially for hemorrhoidal grading higher than G3.

Preoperative and postoperative QoL has emerged as a crucial indicator for assessing the success of surgical procedures. In the context of hemorrhoidal disease, QoL has consistently held significant importance, with numerous studies, including those conducted by Riss et al. [18] and Pastor Peinado et al. [19], designating it as their primary outcome measure. These studies have used the HDSS and the SHS questionnaire during follow-up visits, with a primary focus on evaluating the patient’s well-being.

This emphasis on patient well-being underscores the importance of surgeons in the treatment of hemorrhoidal disease not only prioritizing postoperative outcomes and the prevention of recurrence but also giving due consideration to enhancing the comfort and overall satisfaction of the patient.

The studies from Bussen et al. [21] and Tan et al. [20], instead, explored specifically the long-term outcome and the QoL of surgical management for recurrent hemorrhoidal disease. The 12-month QoL reported by these studies shows that surgical treatment seems to correlate to better QoL compared to the preoperative period. Similar findings arise from our study, following a 24-month follow-up period. After PSM, with both groups having similar preoperative QoL indices, HAL-RAR resulted in a significantly superior postoperative QoL enhancement (0.19 improvement) compared to SH (0.12 improvement). This could be attributed to HAL-RAR’s less invasive and targeted approach.

The strengths of this study are its prospective monocentric nature, the short period considered for the analysis, and the long-term clinical follow-up. We considered the monocentric nature as one of the strengths because the single institution database allowed for greater accuracy in data gathering and analysis.

Some limitations are observed in this study: the small sample size, the considerable discrepancy that may exist among surgeons in assessing the severity of HD, and the stage of each hemorrhoidal pile. Another issue is the uncertainty of the definition of HD recurrence after surgery. In this study, we describe it as the presence at 6 months after surgery of any new symptoms of HD or the persistence of previous ones.

## Conclusions

Both techniques are equally effective in terms of post-operative complications and recurrence rates. Our findings reveal that surgical techniques seem to have little impact on the long-term results when used by well-trained colorectal surgeons. HAL-RAR, however, is a safe and feasible technique in treating grade II-III hemorrhoids showing better outcomes in terms of postoperative pain (during the first 30 days after surgery) and QoL: further analysis with larger patient samples may be needed to confirm these results. In conclusion, future trials should explore the long-term recurrence and reintervention rates of the two procedures. This will help determine the most suitable technique based on preoperative patient assessment, enabling personalized and effective treatment strategies.

## Data Availability

No datasets were generated or analysed during the current study.

## References

[1] Gallo G, Martellucci J, Sturiale A, Clerico G, Milito G, Marino F, Cocorullo G, Giordano P, Mistrangelo M, Trompetto M (2020) Consensus statement of the Italian society of colorectal surgery (SICCR): management and treatment of hemorrhoidal disease Tech Coloproctology 24:145–164 10.1007/s10151-020-02149-1 PMC7005095 31993837

[2] Elmér SE, Nygren JO, Lenander CE (2013) A randomized trial of transanal hemorrhoidal dearterialization with anopexy compared with open hemorrhoidectomy in the treatment of hemorrhoids Dis Colon Rectum 56:484–490 10.1097/DCR.0b013e31827a8567 23478616

[3] Hoyuela C, Carvajal F, Juvany M, Troyano D, Trias M, Martrat A, Ardid J, Obiols J (2016) HAL-RAR (Doppler guided haemorrhoid artery ligation with recto-anal repair) is a safe and effective procedure for haemorrhoids. Results of a prospective study after two-years follow-up Int J Surg 28:39–44 10.1016/j.ijsu.2016.02.030 26876958

[4] Faucheron J-L, Poncet G, Voirin D, Badic B, Gangner Y (2011) Doppler-guided hemorrhoidal artery ligation and rectoanal repair (HAL-RAR) for the treatment of grade IV hemorrhoids: long-term results in 100 consecutive patients Dis Colon Rectum 54:226–231 10.1007/DCR.0b013e318201d31c 21228673

[5] on behalf of the LigaLongo Study Group, Venara A, Podevin J, Godeberge P, Redon Y, Barussaud M-L, Sielezneff I, Queralto M, Bourbao C, Chiffoleau A and Lehur P A (2018) A comparison of surgical devices for grade II and III hemorrhoidal disease. Results from the LigaLongo Trial comparing transanal Doppler-guided hemorrhoidal artery ligation with mucopexy and circular stapled hemorrhoidopexy Int J Colorectal Dis 33:1479–1483 10.1007/s00384-018-3093-8 29808305

[6] Goligher JC (1980) Haemorrhoids or piles. In: Goligher JC (ed) Surgery of the anus, rectum and colon, 4th edn. BAILLIÈRE TINDALL, London, p 96

[7] Dindo D, Demartines N, Clavien P-A (2004) Classification of surgical complications: a new proposal with evaluation in a cohort of 6336 patients and results of a survey Ann Surg 240:205–213 10.1097/01.sla.0000133083.54934.ae PMC1360123 15273542

[8] FallahTafti SP, Foroutani L, Safari R, Hadizadeh A, Behboudi B, AhmadiTafti SM, Keramati MR, Fazeli MS, Keshvari A, Kazemeini A (2023) Evaluation of the Farsi-translated Hemorrhoidal Disease Symptom Score and Short Health Scale questionnaires in patients with hemorrhoid disease: a cross-sectional study. Health Sci Rep 6:e1363 37359414 10.1002/hsr2.1363 PMC10290184

[9] Rørvik HD, Styr K, Ilum L, McKinstry GL, Dragesund T, Campos AH, Brandstrup B, Olaison G (2019) Hemorrhoidal Disease Symptom Score and Short Health ScaleHD: New Tools to Evaluate Symptoms and Health-Related Quality of Life in Hemorrhoidal Disease Dis Colon Rectum 62:333–342 10.1097/DCR.0000000000001234 30451751

[10] Nyström P-O, Qvist N, Raahave D, Lindsey I, Mortensen N (2010) Randomized clinical trial of symptom control after stapled anopexy or diathermy excision for haemorrhoid prolapse Br J Surg 97:167–176 10.1002/bjs.6804 20035531

[11] CarvajalLópez F, Hoyuela Alonso C, Juvany Gómez M, TroyanoEscribano D, TriasBisbal MA, MartratMacià A, ArdidBrito J (2019) Prospective Randomized Trial comparing HAL-RAR versus excisional hemorrhoidectomy: postoperative pain, clinical outcomes, and quality of life Surg Innov 26:328–336 10.1177/1553350618822644 30621513

[12] Van Tol RR, Kleijnen J, Watson AJM, Jongen J, Altomare DF, Qvist N, Higuero T, Muris JWM, Breukink SO (2020) European Society of ColoProctology: guideline for haemorrhoidal disease Colorectal Dis 22:650–662 10.1111/codi.14975 32067353

[13] Karkalemis K, Chalkias PL, Kasouli A, Chatzaki E, Papanikolaou S, Dedemadi G (2021) Safety and effectiveness of hemorrhoidal artery ligation using the HAL-RAR technique for hemorrhoidal disease. Langenbecks Arch Surg 406:2489–2495 33959805 10.1007/s00423-021-02190-0

[14] Krska Z, Kvasnieka J, Faltyn J, Schmidt D, Svab J, Kormanova K, Hubik J (2003) Surgical treatment of haemorrhoids according to Longo and Milligan Morgan: an evaluation of postoperative tissue response Colorectal Dis 5:573–576 10.1046/j.1463-1318.2003.00551.x 14617243

[15] D’Agostino RB (1998) Propensity score methods for bias reduction in the comparison of a treatment to a non-randomized control group Stat Med 17:2265–2281 10.1002/(sici)1097-0258(19981015)17:19<2265::aid-sim918>3.0.co;2-b 9802183

[16] Symeonidis D, Spyridakis M, Zacharoulis D, Tzovaras G, Samara AA, Valaroutsos A, Diamantis A, Tepetes K (2022) Milligan-Morgan hemorrhoidectomy vs. hemorrhoid artery ligation and recto-anal repair: a comparative study. BMC Surg 22(1):416. 10.1186/s12893-022-01861-z. PMID: 36474223; PMCID: PMC9724411 36474223 10.1186/s12893-022-01861-z PMC9724411

[17] Xu L, Chen H, Gu Y (2019) Stapled hemorrhoidectomy versus transanal hemorrhoidal dearterialization in the treatment of hemorrhoids: an updated meta-analysis Surg Laparosc Endosc Percutan Tech 29:75–81 10.1097/SLE.0000000000000612 30540639

[18] Riss S, Weiser FA, Riss T, Schwameis K, Mittlböck M, Stift A (2011) Haemorrhoids and quality of life. Colorectal Dis. 13(4):e48-52. 10.1111/j.1463-1318.2010.02480.x. PMID: 20977590 20977590 10.1111/j.1463-1318.2010.02480.x

[19] Pastor Peinado P, Ocaña J, AbadíaBarno P, Ballestero Pérez A, Pina Hernández JD, Rodríguez Velasco G, Moreno Montes I, MendíaConde E, Tobaruela De Blas E, FernándezCebrián JM, Die Trill J, García Pérez JC (2023) Quality of life and outcomes after rubber band ligation for haemorrhoidal disease. Langenbecks Arch Surg 408:243 37349572 10.1007/s00423-023-02990-6

[20] Tan K-K, Wong J, Sim R (2013) Non-operative treatment of right-sided colonic diverticulitis has good long-term outcome: a review of 226 patients Int J Colorectal Dis 28:849–854 10.1007/s00384-012-1595-3 23070046

[21] Bussen D, Herold A, Bussen S (2012) Gesundheitsbezogene Lebensqualität nach operativer Hämorrhoidaltherapie - Ergebnisse. Methoden und Probleme Zentralblatt Für Chir 137:385–389 10.1055/s-0030-1262703 21294081

